# High-risk coronary artery plaque in asymptomatic patients with type 2 diabetes: clinical risk factors and coronary artery calcium score

**DOI:** 10.1186/s12933-021-01350-2

**Published:** 2021-08-09

**Authors:** Laurits Juhl Heinsen, Gokulan Pararajasingam, Thomas Rueskov Andersen, Søren Auscher, Hussam Mahmoud Sheta, Helle Precht, Jess Lambrechtsen, Kenneth Egstrup

**Affiliations:** 1 Cardiovascular Research Unit, Odense University Hospital, Svendborg Hospital, Baagøes Allé 15, 5700 Svendborg, Denmark; 2 Department of Cardiology, Odense University Hospital, Svendborg Hospital, Baagøes Allé 15, 5700 Svendborg, Denmark

**Keywords:** Coronary computed tomography angiography, Atherosclerosis, Asymptomatic coronary artery disease, High-risk plaque, Coronary artery calcium score, Type 2 diabetes

## Abstract

**Background:**

High-risk coronary artery plaque (HRP) is associated with increased risk of acute coronary syndrome.

We aimed to investigate the prevalence of HRP in asymptomatic patients with type 2 diabetes (T2D), and its relation to patient characteristics including cardiovascular risk factors, diabetes profile, and coronary artery calcium score (CACS).

**Methods:**

Asymptomatic patients with T2D and no previous coronary artery disease (CAD) were studied using coronary computed tomography angiography (CCTA) in this descriptive study. Plaques with two or more high-risk features (HRP) defined by low attenuation, positive remodeling, spotty calcification, and napkin-ring sign were considered HRP. In addition, total atheroma volume (TAV), proportions of dense calcium, fibrous, fibrous-fatty and necrotic core volumes were assessed. The CACS was obtained from non-enhanced images by the Agatston method. Cardiovascular and diabetic profiles were assessed in all patients.

**Results:**

In 230 patients CCTA was diagnostic and 161 HRP were detected in 86 patients (37%). Male gender (OR 4.19, 95% CI 1.99–8.87; p < 0.01), tobacco exposure in pack years (OR 1.02, 95% CI 1.00–1.03; p = 0.03), and glycated hemoglobin (HbA1c) (OR 1.04, 95% CI 1.02–1.07; p < 0.01) were independent predictors of HRP. No relationship was found to other risk factors. HRP was not associated with increased CACS, and 13 (23%) patients with zero CACS had at least one HRP.

**Conclusion:**

A high prevalence of HRP was detected in this population of asymptomatic T2D. The presence of HRP was associated with a particular patient profile, but was not ruled out by the absence of coronary artery calcium. CCTA provides important information on plaque morphology, which may be used to risk stratify this high-risk population.

*Trial registration* This trial was retrospectively registered at clinical trials.gov January 11, 2017 trial identifier NCT03016910.

## Introduction

Acute myocardial infarction (MI) is mainly caused by the rupture of a coronary artery plaque and subsequent coronary artery occlusion. Ruptured plaques are characterized as lipid-rich plaques with positive remodeling (PR) covered by a thin fibrous cap infiltrated by inflammatory cells [1, 2]. Beyond the assessment of coronary artery stenosis coronary computed tomography angiography (CCTA) enables evaluation of coronary artery plaque morphology and burden. Mounting evidence suggests, that plaque morphology assessed by CTTA [3], and by invasive coronary imaging [4, 5], is an important prognostic factor. In particular, plaques with both low attenuation and positive remodeling have a strong association with cardiac events, and may be considered high-risk plaques (HRP) [6–8]. However, the characteristic of patients with high-risk plaque and the relation to atherosclerotic burden is less clear.

Large epidemiological studies have demonstrated that patients with type 2 diabetes (T2D) and no prior MI have the same cardiovascular risk as non-diabetics with a previous MI [9, 10]. Furthermore, patients with T2D have a 30% greater mortality when suffering a MI compared to patients without T2D [11]. Despite effective risk-factor control, individuals with T2D still have twice the risk of MI as non-diabetics [11]. This residual risk of MI implies that intrinsic properties of diabetes are involved in the atherosclerotic process. However, the mechanisms underlying the increased frequency of MI in T2D are not yet well understood. The objectives of this study were the following: (1) to examine the prevalence of HRP in asymptomatic T2D; (2) to investigate the relationship between HRP and cardiovascular risk factors and diabetes profile; (3) to investigate the relationship between HRP and the coronary artery calcium score (CACS).

## Methods

### Study population

This was a single-center cross-sectional study performed at Odense University Hospital, Svendborg, Denmark, from March 2016 to September 2017. A total of 261 consecutive patients with asymptomatic T2D, over the age of 18, gave written informed consent for participation in the study. There was no clinical indication for CCTA, and the examination was exclusively performed with the purpose of a descriptive study. Patients were recruited at the Endocrinology Outpatient Clinic and the hospital Retina Photography Clinic. The diagnostic criteria for type 2 diabetes included hbA1c ≥ 48 mmol/mol on two separate occasions and the absence of glutamatic acid decarboxylase antibodies [12]. Patients were not eligible if they had a history of coronary artery disease (CAD), documented heart failure, reduced glomerular filtration (< 45 ml/min), or allergy to iodine contrast. A detailed health status was recorded in all patients. Blood was drawn for creatinine, glycated hemoglobin (HbA1c), lipid profile, CRP and troponin T. All patients had a spot urine was collected to assess micro- and macroalbuminuria. Albuminuria was defined as albumin-creatinine ratio ≥ 30 mg/L in at least two independent samples and no symptoms of urinary tract infection. Blood pressure was measured in a seated position after at least 10 min of rest. Patients receiving one or more antihypertensive drugs were categorized as having hypertension. Patients treated with one or more lipid-lowering drugs were categorized as having hypercholesterolemia. Patients underwent annual screening for retinopathy and neuropathy as a part of their clinical controls. Retinopathy was graded from 0 to 4 [13], and peripheral neuropathy was graded as normal, reduced, or absent. Diabetic complications were defined as presence of any degree of albuminuria, retinopathy, and/or neuropathy. Smoking history was evaluated to assess the total tobacco exposure in terms of pack years (1 pack year = 20 cigarettes per day for one year) [14].

### CTTA acquisition

The CCTA images were acquired using a standardized protocol for a 256-detector system (GE-revolution CT, GE healthcare, Waukesha, Wisconsin, USA). An unenhanced scan was initially performed to assess coronary artery calcium.

Patients were prepared for the scan with tablet ivabradine 7.5 mg once daily two days prior to the scan. If needed, intravenous beta blocker was administered on the day of the scan in patients with increased heart rate (> 65 beats per minute) [15]. Sublingual fast acting nitrates were administered just before the enhanced scan. Images were obtained by an ECG-gated prospective acquisition in the 75% of the R-R interval with an additional padding of 45 ms (ms) to allow additional reconstruction. In patients with a high heart rate an additional phase was acquired in the 40% phase of the R-R interval, and a repeated scan was acquired if the heart rhythm was irregular. A total of 60 ml of iodine contrast (Visipaque 320 mg iodine/ml) was injected at 5 ml/s, and the scan was timed visually, when maximal attenuation was detected in the ascending aorta. Tube voltage and current were modulated to the patient’s body size with a tube voltage between 80 to 140 kV and a tube current between 150 and 700 milliampere according to body mass index (BMI). Gantry rotation time was 280 ms with 16 cm axial coverage. The median radiation dose per scan was 2.01 millisievert.

The slice thickness and interval for reconstruction were both 0.625 mm, and 40% adaptive statistical iterative reconstruction (ASiR) was adopted. All available phases were reconstructed, and images with superior image quality were selected for analysis.

### Quantitative CCTA analysis

Reconstructed images were analyzed on an offline workstation with validated semiautomatic software (Qangio CT Research Edition ver. 3.1.3.18, Medis NL) [16]. Experienced observers (LJH and TRA) analyzed all images while blinded to patient characteristics. Plaque location was designated according to the American Heart Association modified 17 segment model [17]. Centerlines of each coronary artery were automatically extracted by the software. On the basis of longitudinal images, cross sectional lumen and vessel contours were created and manually corrected by the observer if necessary. Segments with insufficient image quality were excluded, and the software automatically excluded segments with a lumen diameter less than 1.5 mm. Diagnostic CCTA was considered as an overall image quality allowing qualitative and quantitative plaque analysis. Patients with severe motion artifacts or otherwise compromised image quality were excluded from the analysis.

All available segments were screened for the presence of plaque, and segments without visible plaque were excluded from the analysis [18]. For each patient the following parameters were calculated: total atheroma volume (TAV) (total vessel volume—total lumen volume), percent atheroma volume (PAV) (total vessel volume—total lumen volume/total vessel volume × 100%), and normalized atheroma volume (NAV) (total vessel volume—total lumen volume/mean segment length).

Furthermore, the volumes of plaque types dense calcium, fibrous, fibrous–fatty, and necrotic core were calculated on the basis of Hounsfield Units (HU). The software applied a dynamic algorithm that adapted the HU thresholds according to luminal contrast densities [19]. To address the differences in length of vessels analyzed in HRP and non-HRP patients TAV and volumes of fibrous, fibrous-fatty, necrotic core and necrotic core plaque were normalized according to vessel length by the following formula: (plaque volume/segment length) × mean segment length population.

### Qualitative CCTA analysis

All non-calcified and mixed plaques were screened for the following plaque features:

1. Low attenuated plaque (LAP): non-calcified or mixed plaque with a volume > 1 mm^3^ containing voxels with attenuation less than 30 HU.
2. Positive remodeling (PR): Remodeling indices was calculated as lesion diameter/reference area, where the reference diameter is from a normal/minimal diseased proximal segment. PR was defined as more than 5% area remodeling [20, 21].
3. Spotty calcification (SC): Calcified elements less than 3 mm in length occupying less than 90 degrees of the coronary arc.
4. Napkin ring sign (NRS): A non-calcified ring like structure surrounding a central area with a lower attenuation. HRP was defined as a plaque with the presence of two or more high-risk features.

Furthermore, for all plaque the percent diameter stenosis was calculated by the following: 1 − (minimal lumen diameter plaque/lumen diameter reference segment) × 100%. The degree of stenosis were reported as mild (25–49%), moderate (50–69%) and severe stenosis (> 70%).

### Coronary artery calcium score

The coronary artery calcium score (CACS) was calculated by the Agatston score using the unenhanced CT images. Patients were categorized in groups according to the CACS:

No CAD (CACS = 0), minimal CAD (CACS = 1–10), mild CAD (CACS = 11–100), moderate CAD (CACS = 101–400), and severe CAD (CACS > 400).

### Statistical analysis

Statistical analyses were performed by STATA IC 14.2. Baseline parameters were presented as means with standard deviation. Risk factors with a non-normal distribution were presented as medians with interquartile range. A t-test was performed to assess the relationship between baseline parameters and HRP on a per-patient level. The association of HRP and variables with a p < 0.05 were tested in univariate and multivariate logistic analysis adjusted for confounders including age, sex, dyslipidemia, and systolic blood pressure. Inter- and intra-reader agreement were assessed by Pearson’s correlation coefficient (Pearson’s r) and Bland–Altman analysis using 95% limits of agreement (LOA) [22] for twenty random vessels.

## Results

### Study population

Of the 261 patients recruited for the study, 230 had a diagnostic CCTA and were included in the final analysis. Mean age was 62 ± 10 years and 73% of patients were men. The mean diabetes duration was 11 ± 8 years, and 54% of the patients had diabetic complications. Retinopathy was present in 52 patients (stage 1; n = 23, stage 2; n = 21, stage 3: n = 3, stage 4: n = 5). Albuminuria was present in 65 patients (microalbuminuria n = 61 and macroalbuminuria n = 4.) and neuropathy was present in 57 patient and 38 patients (17%) had two or more complications. In 144 (63%) of patients, diabetes care was managed by a specialist at the Endocrinology Outpatient Clinic, and the remaining patients were followed by their general practitioner (GP). We identified 161 high-risk plaques in 86 (37%) patients. Furthermore, we identified CAD without HRP in 117 (51%) patients, and 27 (12%) patients had no evidence of CAD.

### Cardiovascular risk factors

High-risk plaques were more frequent in men (90 vs. 63%, p < 0.001) and were associated with a higher tobacco exposure in pack years (20.2 vs. 13.1, p = 0.001), and patients with HRP had a lower BMI (29.4 vs. 31.2 kg/m^2^, p = 0.001) (Table 1). In the univariate logistic regression male gender (OR 4.92, 95% CI: 2.28–10.6, p < 0.001) and smoking in pack years (OR 1.02, 95% CI: 1.00–1.03, p = 0.01) were significant predictors of HRP and remained significant in the multivariate analysis. BMI was negatively associated with HRP (OR 0.92, 95% CI: 0.86–0.97, p = 0.01) but was not significant in the multivariate analysis (Table 2). Baseline characteristics were compared between men and women and men had a greater tobacco exposure (17.5 ± 20.1 vs. 11.6 ± 15.9 pack years, p = 0.04). In addition, men had higher HbA1c (61.5 ± 14.4 vs. 55.9 ± 12.7 mmol/mol, p = 0.01), and a greater proportion of diabetic complications (64 vs. 47%, p = 0.01). The median CACS was higher in men (137.0 IQR: 13–699 vs. 4.0 IQR 0–121, p < 0.001). Baseline characteristics stratified by sex are shown in Table 5 in the appendix section.

**Table 1:** Baseline characteristics of patients with and without high-risk plaque

Variable	Overall n = 230	HRP present n = 86	HRP not present = 144	p-value
Age	62 ± 10	62 ± 9	61 ± 10	0.40
Male, n (%)	144 (63)	77 (90)	91 (63)	** < 0.001**
BMI, kg/m^2^	30.6 ± 3.78	29.4 ± 3.78	31.23 ± 5.17	** 0.01**
Waist circumference, cm	107 ± 15	106 ± 9	107 ± 15	0.47
Waist / hip –ratio	1.00 ± 0.1	1.02 ± 0.1	0.99 ± 0.1	**0.02**
Outpatient diabetes care, n (%)	144 (63)	52 (60)	92 (64)	0.56
Hypertension, n (%)	160 (70)	57 (66)	102 (71)	0.52
Hypercholesterolemia, n (%)	179 (78)	65 (76)	114 (79)	0.53
Current smoker, n (%)	53 (23)	25 (29)	27 (19)	0.07
Pack years	15.9 ± 19.2	20.2 ± 22.7	13.1 ± 16.2	** 0.001**
Systolic blood pressure, mmHg	141.3 ± 15.7	141.5 ± 15.8	141.2 ± 15.3	0.87
Diastolic blood pressure, mmHg	85.8 ± 10.2	86.6 ± 9.74	85.4 ± 10.53	0.38
Laboratory findings				
LDL cholesterol, mmol/L	2.0 ± 0.8	2.1 ± 0.8	1.9 ± 0.8	0.15
HDL cholesterol, mmol/L	1.3 ± 0.7	1.2 ± 0.5	1.3 ± 0.7	0.97
Triglycerides, mmol/L	2.0 ± 1.1	2.2 ± 1.2	2.0 ± 1.1	0.21
CRP, mg /L	2.9 ± 3.7	2.6 ± 3.7	3.0 ± 3.7	0.35
Troponin, T ng/L	10.0 ± 8.6	10.8 ± 9.8	9.5 ± 7.8	0.28
HbA1c, mmol/mol	60.1 ± 14.1	64.1 ± 15.8	57.7 ± 12.6	** < 0.001**
CACS, median [IQR]	72.5 [1;460]	113.0 [25;408]	28.5 ± [0;612]	0.11
Duration of diabetes, years	10.9 ± 8.0	10.1 ± 9.0	11.4 ± 7.3	0.21
Lipid-lowering duration, years	5.3 ± 5.7	5.1 ± 5.7	5.5 ± 5.7	0.58
Diabetic complications, n (%)	123 (54)	55 (64)	68 (47)	**0.01**
Retinopathy, n (%)	52 (23)	25 (29)	27 (19)	0.07
Albuminuria, n (%)	65 (28)	30 (35)	35 (24)	0.09
Neuropathy, n (%)	57 (25)	23 (27)	34 (24)	0.28
Medication, n (%)				
Lipid-lowering, n (%)	178 (77)	65 (76)	113 (78)	0.61
Aspirin, n (%)	30 (13)	10 (12)	21 (15)	0.30
Beta blocker, n (%)	23 (10)	5 (6)	18 (13)	0.10
ACE/ARB inhibitor, n (%)	152 (66)	56 (65)	96 (67)	0.81
Insulin, n (%)	90 (39)	28 (33)	62 (43)	0.11
GLP-1 inhibitor, n (%)	54 (23)	15 (17)	39 (27)	0.10
DPP4 inhibitor, n (%)	35 (15)	17 (20)	18 (13)	0.14
Biguanide, n (%)	192 (83)	77 (90)	115 (80)	0.06
Sulfonylurea, n (%)	40 (17)	21 (24)	19 (13)	**0.03**
SGLT2, n (%)	21 (9)	9 (10)	12 (8)	0.59

Baseline characteristics were compared between patients with and without HRP. A p < 0.05 was considered a significant

*BMI* body mass index, *ACE/ARB* ace converting enzyme inhibitor/angiotensin receptor blocker, *LDL* low-density lipoprotein, *HDL* high density lipoprotein, *CRP* c-reactive protein, *CACS* coronary artery calcium score, *GLP-1* glucagon like peptid-1, *DPP4 inhibitor* dipeptidyl peptidase-4 inhibitors, *SGLT-2* sodium glucose cotransport-2 inhibitor

**Table 2:** Multivariate analysis of predictors for high-risk plaque. N = 230

Variable	Univariate			Multivariate		
	OR	95% CI	p-value	OR	95% CI	p-value
Male	4.92	2.28–10.6	** < 0.001**	4.19	1.99—8.87	** < 0.001**
BMI	0.92	0.86–0.97	**0.01**	0.93	0.86–1.00	0.06
Pack Years	1.02	1.00–1.03	** 0.01**	1.02	1.00–1.03	**0.03**
Any diabetic complication	1.95	1.13–3.38	**0.02**	1.74	0.97–3.10	0.06
HbA1c	1.03	1.01–1.05	** 0.001**	1.04	1.02–1.07	** 0.01**
Sulfonylureas	2.14	1.08–4.27	**0.03**	1.98	0.96–4–07	0.06

In the multivariate analysis predictors were adjusted for age, sex, hypercholesterolemia, and systolic blood pressure

*HbA1c* Hemoglobin A1c

### Diabetes-specific factors

Patients with HRP had 11% higher HbA1c levels compared to patients without HRP (64.1 vs. 57.7 mmol/mol, p = 0.01). The presence of any diabetic complication (retinopathy, albuminuria, and neuropathy), was more frequent in patients with HRP (64% vs. 47%, p = 0.01) and patients with HRP were more frequent sulfonylurea users (24 vs. 13%, p = 0.03) (Table 1).

In univariate analysis, Hb1Ac (OR 1.03, 95% CI: 1.01–1.05, p = 0.001), any diabetic complication (OR 1.95, 95% CI: 1.13- 3.38, p = 0.02), and use of sulfonylureas (OR 2.14, 95% CI: 1.08–4.27, p = 0.03) were significant predictors of HRP, but only HbA1c remained statistically significant in the multivariate analysis (Table 2).

### Coronary artery calcium score

**Fig. 1:**
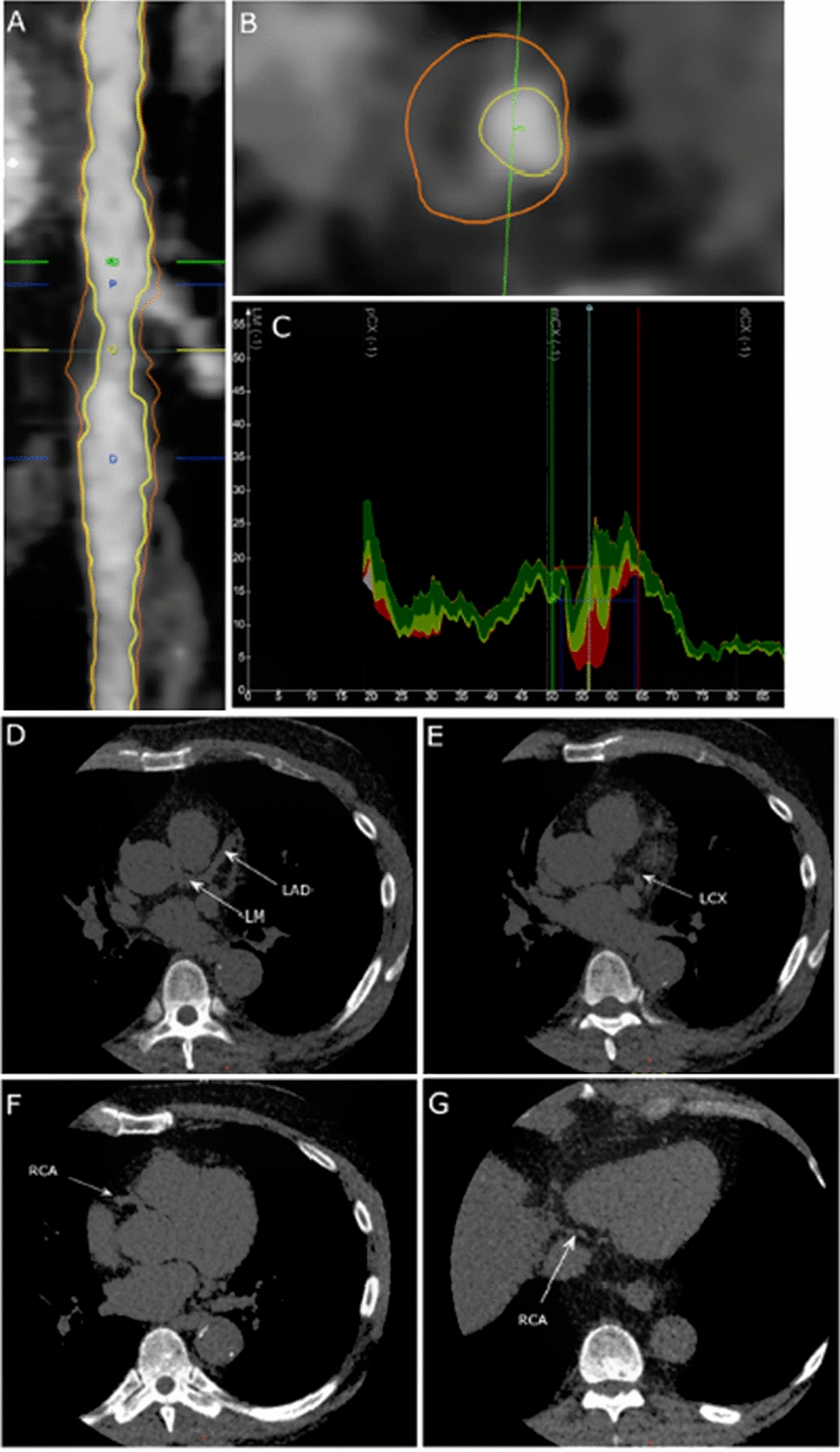
Plaque analysis with semiautomatic plaque analysis software. **A** Longitudinal straightened multiplanar reconstruction of left circumflex artery featuring a high-risk plaque (HRP) between the blue lines. **B** Transverse vessel view demonstrating HRP with positive remodeling, napkin ring sign, and low attenuation core (< 30 HU). **C** Graph depicting lumen and vessel areas as a function of vessel length. Plaque subtypes dense calcium, fibrous, fibrous-fatty and necrotic core are shown in grey, green, light green, and red colors. The bottom panels shows unenhanced axial images in the same patient demonstrating no coronary artery calcium present in the left main (LM) and left descending artery (LAD) (**D**), left circumflex artery (LCX) (**E**), and right coronary artery (RCA) (**F, G**)

**Fig. 2:**
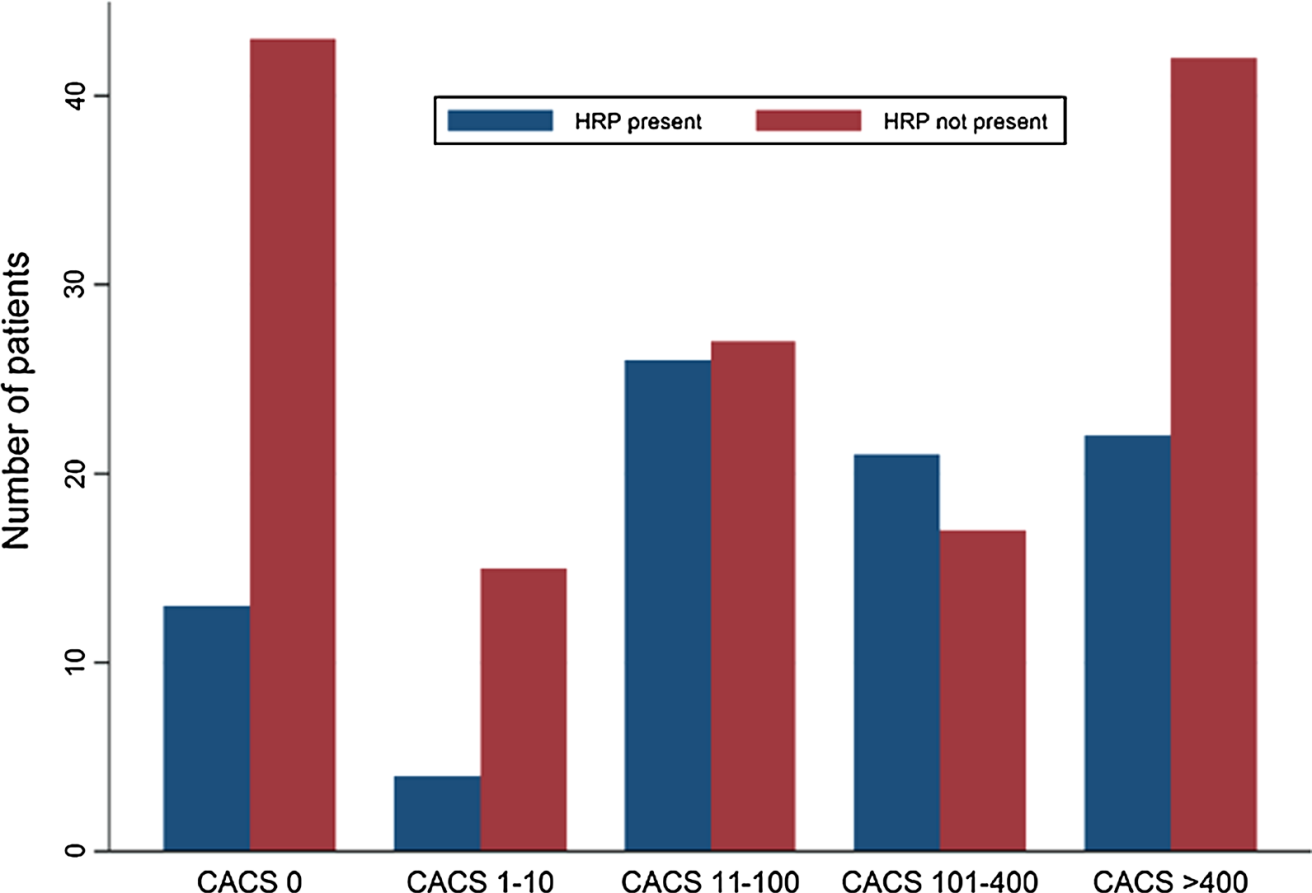
Distribution of 230 patients with and without high-risk plaque (HRP) stratified by coronary artery calcium score (CACS)

Median CACS was 75.5 (IQR: 1.00–460), and HRP was detected in all CACS groups. We identified 13 patients (23%) with HRP and CACS = 0 (Fig. 1). The highest frequency of patients with HRP was found in patients with moderate calcium score (CACS = 11–100) (Fig. 2). In the group of patients with HRP and no or minimal CACS fewer used lipid-lowering medication (59 vs. 80%, p = 0.07) and the duration of lipid-lowering treatment was significantly shorter compared to patients with HRP and mild to severe CACS (2.3 ± 3.2 vs. 5.7 ± 6.0 years, p = 0.03).

### CCTA findings

The measures of plaque burden stratified by the presence of HRP are presented in Table 3.

**Table 3:** Total atheroma volume and plaque composition in patients with CAD with HRP and CAD without HRP. N = 203

Variable	CAD with HRP n = 86	CAD without HRP n = 117	p-value
Vessel length, mm	209.1 ± 109.0	187.5 ± 117.7	0.18
TAV, mm^3^	867.5 ± 471.1	782.7 ± 653.1	0.31
PAV (%)	34.4 ± 7.0	31.9 ± 10.1	0.05
Normalized TAV	745.9 ± 250.6	730.7 ± 328.7	0.71
-Fibrous volume, mm^3^	432.2 ± 152.0	449.6 ± 169.4	0.45
-Fibrous-fatty volume, mm^3^	142.2 ± 63.5	108.4 ± 61.6	** < 0.001**
-Necrotic core volume, mm^3^	104.5 ± 100.1	43.8 ± 76.3	** < 0.001**
-Dense calcium volume, mm^3^	64.0 ± 69.4	131.1 ± 190.5	**0.002**
Coronary stenosis			
Mild (25–49%), n (%)	68 (79)	75 (64)	**0.01**
Moderate (50–69%), n (%)	32 (37)	30 (26)	**0.01**
Severe (≥ 70%), n (%)	3 (3)	3 (3)	0.52

Volumes of fibrous, fibrous-fatty, necrotic core and necrotic core plaque were normalized according to vessel length

*TAV* total atheroma volume, *PAV* percent atheroma volume, *NAV* normalized atheroma volume

In 27 patients we did not find any evidence of coronary atherosclerosis, and they were excluded from this analysis. There was no significant difference in the length of vessels analyzed between groups (187.5 ± 117.7 mm vs. 209.1 ± 109.0 mm; p = 0.183). Patients with HRP did not have significantly higher TAV compared to patients without HRP (787.7 ± 653.1 mm^3^ vs. 867.5 ± 471 mm^3^; p = 0.18). There was no difference in the other quantitative plaque measures PAV and normalized TAV between groups either. However, patients with HRP had significantly larger normalized volumes of fibrous-fatty (142.2 ± 63.5 mm^3^ vs. 108.4 ± 61.6 mm^3^; p < 0.001) and necrotic core volumes (104.5 ± 100.1 mm^3^ vs. 43.8 ± 76.3 mm^3^; p < 0.001), while patients with no HRP presented significantly larger volumes of dense calcium (64.0 ± 69.4 mm^3^ vs. 131.1 ± 190.5 mm^3^; p = 0.002). Mild stenosis were found more frequent in patients with HRP (68 vs 75%; p = 0.01) as well as moderate stenosis (37 vs 26%; p = 0.01). Severe stenosis were found in three patients with HRP and three patients without high-risk plaque. Percent diameter stenosis could not be calculated in 19 without HRP due to extensive calcification. This was only the case in one patient with HRP.

### Inter- and intra-observer variability

Repeatability was good, and the mean bias for intra-observer and inter-observer variability for total plaque were 5.0 mm^3^, 95% LOA (− 89.7; 99.7) and 8.5 mm^3^, 95% LOA (− 176.1; 193.1), respectively. A strong correlation was detected between both intra-observer analysis (r = 0.98) and inter-observer analysis (r = 0.91). Inter- and intra-observer agreement and correlation for additional plaque components are shown in Table 4.

**Table 4:** Inter- and intra-observer variability

Variable	Intra-observer variability		Inter-observer variability	
	Mean diff. [95% LOA]	r	Mean diff. [95% LOA]	r
Total plaque volume	5.0 [− 89.7; 99.7]	0.98	8.5 [− 176.1; 193.1]	0.91
Dense plaque	1.6 [− 22.9; 26.1]	0.99	0.5 [− 28.9; 29.9]	0.99
Fibrous plaque	5.9 [− 11.5; 23.2]	0.95	5.7 [− 13.5; 24.9]	0.95
Fibrous-fatty plaque	1.1 [ − 28.7; 30.9]	0.96	5.3 [− 44.9; 55.4]	0.87
Necrotic core plaque	− 3.9 [− 45.7; 37.9]	0.91	− 3.1 [− 55.7; 49.4]	0.86

*Mean diff* Mean difference between paired observations, *LOA* Limits of agreement calculated as the mean difference ± 1.96 standard deviation; r: Pearson’s coefficient of correlation between paired observations

## Discussion

The main findings of this study are the following: (1) HRP was identified in 86 asymptomatic patients with T2D. (2) Men were more likely to have HRP compared to women. (3) HbA1c was the only diabetes-specific risk factor which remained significantly associated with HRP. (4) HRP was most frequently seen in patients with “mild CAD” (CACS = 11–100) but was identified in all strata including patients with “No CAD” (CACS = 0).

A limited number of studies have assessed plaque morphology in asymptomatic diabetes using varying definitions of HRP. Using a similar definition of HRP as our study, Kamimura et al. reported that HRP was only present in 17% of patients with asymptomatic diabetes but included patients with lower BMI and hb1Ac compared to our study [23]. Nezarat et al. examined subclinical CAD in patients with diabetes younger than 40 years and reported that non-calcified plaque were present in 28% of the population [24]. In a population similar to the present study, Halon et. al. found 376 high-risk plaque in 499 patients with asymptomatic diabetes [25]. This number HRP seems to be comparable to our study though using a slightly different definition of HRP. In the study by Halon, events during 9-years follow-up were reported and the authors found that the majority of events were caused by HRP while events in non-HRP were rare. The risk of acute events in the study increased by the number of high-risk plaque features, but also with the degree of stenosis. In addition, more densely calcified plaque was considered a protective factor.

In our study, male gender was associated with a four time greater odds of having a HRP compared to females, which could indicate a higher risk for males. This finding contradicts several meta-analyses that have demonstrated that diabetes equalizes the risk of CAD between men and women [26, 27]. We detected several differences in the risk profile between men and women. Men had a greater exposure to smoking, a higher HbA1c, and a higher burden of late diabetic complications compared to women, which could in part explain the higher proportion of HRP in men. Another explanation could be the differences in plaque phenotypes between men and women observed in autopsy studies. In patients with sudden cardiac death from coronary thrombosis, plaque erosion is more common in women than in men [28, 29]. In plaque erosion, the plaques tend to be more rich in smooth muscle cells, lack the superficial lipid core, and tend to be less calcified [30]. Because of these morphological differences, plaques prone to erosion may not be detected as HRP by CCTA explaining an underestimation of HRP in women.

Smoking is a well-established risk factor for cardiovascular events. The exact role of smoking in plaque formation is less clear. In observational studies using intravascular ultrasound or optical coherence tomography, smoking was associated with increased lipid content in coronary plaque in patients with symptomatic CAD [31–33]. A strong association between the number of cigarettes smoked in pack years and the presence of HRP was found. We suggest, that smoking may have an important role in the formation of high-risk plaques, and that pack years may be a superior risk marker compared to smoking as simple dichotomous risk factor.

We examined several diabetes-specific factors including HbA1c, diabetes duration, and presence of any diabetic complications in relation to HRP. The only significant association was found between HbA1c and HRP. It has been demonstrated in large registry trials, that increased levels of fasting blood glucose is associated with increased risk of coronary heart disease [11]. However, several studies intervening on blood glucose levels to improve patient-outcome have failed [34–36]. In observational studies higher levels of HbA1c have been associated with an increased lipid content in coronary plaques [37] and impaired regression of atheroma volume during statin therapy [38]. In addition, autopsy studies have demonstrated that HbA1c is an independent predictor of necrotic core size [39]. In our study patients with HRP were well treated with a mean low-density lipoprotein cholesterol of 2.1 mmol/L. However, triglycerides and triglyceride-rich lipoproteins, associated with the metabolic syndrome, T2D and insulin resistance, are less affected by statin treatment [40]. These highly atherogenic lipoproteins may account for some of the residual risk in T2D with well-treated LDL-cholesterol and could explain the association of higher hbA1c in patients with HRP. The coronary artery calcium score (CACS) has been proposed as a relevant method to risk stratify asymptomatic high-risk patients due to widespread availability and low cost. While large registries like the MESA-study [41] found higher CACS in patients with diabetes more recent propensity matched studies did not find any difference in the extent of coronary artery calcium in patients with T2D compared to patients without T2D[42, 43]. In addition, several studies using CCTA found non-calcified plaque in patients with zero CACS [23, 44]. In our study HRP was most frequently seen in patients with “mild CAD” (CACS = 11–100). More concerning, in 23% of the patients with zero CACS. HRP was present. These findings are similar to the findings by Min et. al. how found that atherosclerosis was present in one third of asymptomatic patients with diabetes a CAS of zero, and that 10% of these patients had obstructive CAD [45]. In patients with no or minimal CACS who had HRP, the duration of lipid-lowering treatment was significantly shorter, and fewer of these patients were in fact treated with lipid-lowering medication. This finding may also reflect that the early atherosclerotic plaque formation does not involve calcification, or at most microcalcifications, that does not show on CT. The increased risk of acute events arising from non-calcified plaque compared to calcified plaque [3] suggests that the progressive plaque calcification could be a stabilizing factor. In this respect, statins may play an important role facilitating plaque stabilization through plaque calcification [46, 47].Despite these findings, the incidence of events in asymptomatic T2D with no CAD defined by CACS has been very low in several prognostic studies [48, 49]. However, the utility of CACS as screening tool in asymptomatic patients with diabetes has not yet been examined in a randomized trial and should therefore be used with caution.

The atherosclerotic disease burden is a well-established predictor of major cardiovascular events in patients with established CAD [50, 51]. It could be hypothesized that patients with HRP have an increased risk due to a greater global disease burden rather than from the HRP itself. Our results did not demonstrate a significant difference in TAV between patients with and without HRP. However, there was a significant difference in plaque composition, and patients with HRP had larger volumes of non-calcified plaques and less dense calcium, which could be an indicator of more unstable plaques [46]. Overall, our findings suggest that HRP could be an important feature of coronary artery disease independent of the global disease burden.

Asymptomatic diabetes is a very heterogeneous group, and the challenge is finding the true high-risk patients. There were no evidence of CAD in 12% of the patients in our study, which indicate an excellent prognosis and a low risk of cardiovascular events. CCTA screening has not yet demonstrated any prognostic benefits. However, there will be in increased demand for individualized treatment, and CCTA could play an important role in the future. In the PROSPECT ABSORB trial, preventive percutaneous coronary intervention (PCI) of non-culprit, non-obstructive high-risk lesions were neither beneficial nor hazardous compared to medical treatment. Sealing of high-risk plaque using a coronary stent may be a way forward but larger studies are needed to demonstrate clinical endpoints [52]. It has been suggested that coronary artery plaque undergo rapid progression prior to MI [53]. Accepting this thesis, lipid control becomes paramount as cholesterol levels is the major modifiable factor in plaque progression. Emerging pharmacological therapies with effects beyond statins are becoming available and allow for an escalation of treatment in patients with HRP and could be the focus of future randomized trials.

The findings in this study as well as new markers of high-risk plaque morphology need to be validated in larger trials. This could lead to a better selection of asymptomatic individuals with diabetes who could benefit from CCTA screening.

### Limitations

This was a small observational study and is therefore prone to confounding and bias by design. We did not include a control group to compare asymptomatic patients with diabetes to individuals without diabetes. Furthermore, we did not report any prognostic information to back up the claim, that HRP is in fact associated with cardiovascular events, although this point has been supported by several trials in recent years. A general problem in plaque morphology studies is the lack of consensus on how to define high-risk plaque, which makes comparison of results between studies problematic. Finally, the proportion of patients with evidence of CAD and HRP was high in our study compared to other studies of asymptomatic diabetes [23, 54] which could indicate that this was in fact a selected high-risk population. The majority of patients were followed at the Endocrinology Outpatient Clinic rather than by their GP, which could reflect that these patients were more complex and challenging to treat. For this reason, the findings of the present study should be interpreted with caution. A major strength of this study is the thorough collection of patient characteristics collected “hands on” by the authors, which increases the reliability and uniformity of data compared to register based data.

## Conclusions

We found a high frequency of high-risk plaques in asymptomatic patients with T2D despite effective lipid-lowering treatment. HRP was significantly associated with male gender, increased number of pack years, and an increased HbA1c while the absence of coronary artery calcium did not exclude HRP. We believe CCTA could be used to risk stratify asymptomatic patient with T2D based upon the presence of HRP. Whether this improves patient outcome needs further investigation.

## Data Availability

The datasets used and/or analysed during the current study are available from the corresponding author on reasonable request.

## Appendix 1

See Table 5.

**Table 5:** Baseline characteristics stratified by sex

Variable	Overall n = 230	Men n = 168	Women n = 62	p-value
Age	62 ± 10	62 ± 10	61 ± 9	0.37
BMI, kg/m^2^	30.6 ± 4.8	31.4 ± 5.8	30.3 ± 4.3	0.09
Hypertension, n (%)	160 (70)	43 (66)	117 (71)	0.97
Hypercholesterolemia, n (%)	179 (78)	128 (76)	51 (82)	0.34
Current smoker, n (%)	53 (23)	41 (24)	12 (19)	0.42
Pack years	15.9 ± 19.2	17.5 ± 20.1	11.6 ± 15.9	**0.04**
Laboratory findings				
LDL cholesterol, mmol/L	1.99 ± 0.81	1.94 ± 0.82	2.12 ± 0.78	0.13
HDL cholesterol, mmol/L	1.3 ± 0.7	1.2 ± 0.7	1.3 ± 0.4	0.37
HbA1c, mmol/mol	60.0 ± 14.4	61.5 ± 14.4	55.9 ± 12.7	** 0.01**
CACS, median [IQR]	72.5 [1;460]	137.0 [13;699]	4 0.0 [0;121]	** < 0.001**
Duration of diabetes, years	10.9 ± 8.0	11.1 ± 8.2	10.4 ± 7.4	0.55
Lipid-lowering duration, years	5.3 ± 5.7	5.3 ± 5.8	5.4 ± 5.4	0.86
Diabetic complications, n (%)	123 (54)	55 (64)	68 (47)	**0.01**
Medication				
Lipid-lowering, n (%)	178 (77)	127 (76)	51 (82)	0.28
Insulin, n (%)	90 (39)	69 (41)	21 (52)	0.32

Baseline characteristics were compared between men and women. A p < 0.05 was considered significant

*BMI* body mass index, *LDL* low density lipoprotein, *HDL* high density lipoprotein, *CACS* coronary artery calcium score.

## Abbreviations

MI: Acute myocardial infarction
CAD: Coronary artery disease
T2D: Type 2 diabetes mellitus
CCTA: Coronary computed tomography angiography
HRP: High-risk plaque
LAP: Low-attenuated plaque
PR: Positive remodeling
CACS: Coronary artery calcium score
